# Exploring the Public Awareness of Thyroid Cancer in Northern Saudi Arabia: A Preliminary Stage for Health Promotion

**DOI:** 10.3390/healthcare13111289

**Published:** 2025-05-29

**Authors:** Yousef Saleh Alalawi, Ryanh Hamoud Alanazi, Hadeel Thamer Alanazi, Nouf Khalid J. Alanazi, Aljawharah Abdulrahman Alrayes, Raghad Fahad Alruwaili, Wjood Fahad Alanazi, Manal S. Fawzy

**Affiliations:** 1 Department of Surgery, King Salman Armed Forces Hospital, Tabuk 47512, Saudi Arabia; yalalawi@hotmail.com; 2 College of Medicine, Northern Border University, Arar 73213, Saudi Arabia; rayanhalenazi10@gamil.com (R.H.A.); dr.hadeel0509@gmail.com (H.T.A.); dr.nuofal3nazi@gmail.com (N.K.J.A.); 3 College of Medicine, Aljouf University, Sakakh 72388, Saudi Arabia; aljawharah.alrayes28@gmail.com; 4 College of Medicine, Hail University, Hail 81422, Saudi Arabia; s201907666@uoh.edu.sa (R.F.A.); s201907660@uoh.edu.sa (W.F.A.); 5 Center for Health Research, Northern Border University, Arar 73213, Saudi Arabia

**Keywords:** thyroid cancer, awareness, knowledge, Saudi Arabia, risk factors, prevention, early detection, public health

## Abstract

Background/Objectives: Thyroid cancer is the most common endocrine malignancy in Saudi Arabia, with rising incidence and notable gender disparities. However, public awareness and understanding, particularly in the Northern region, remain limited. This study aims to assess knowledge, awareness, and preventive practices regarding thyroid cancer among northern Saudi Arabian residents and identify sociodemographic factors associated with levels of knowledge, awareness, and engagement in preventive practices. Methods: A cross-sectional survey of 702 participants from northern Saudi Arabia was conducted using a validated online questionnaire. Participants were recruited via social media platforms and online community groups. The survey assessed sociodemographic data, knowledge of risk factors, symptoms, and screening practices, perceptions of curability and prevention, and engagement with awareness campaigns. Descriptive statistics and chi-square tests were used to analyze associations. Results: The majority of respondents were young, female, and highly educated. Only 1.3% reported a personal history of thyroid cancer. Knowledge gaps regarding risk factors, screening practices, and curability were evident: 54.6% had never undergone thyroid hormone analysis, and 91% had not received thyroid imaging. Nearly half (48.6%) were uncertain about the curability of thyroid cancer, and only 27.8% recognized its genetic basis. While 62.1% believed thyroid cancer could be prevented, just 18.4% participated in awareness campaigns. Significant associations were found between knowledge of screening practices and age, education, nationality, and having family or friends in the medical field (*p* < 0.05). Conclusions: Significant gaps in awareness of thyroid cancer risk factors and early detection practices exist among northern Saudi residents. Culturally tailored educational interventions and integration of thyroid health into primary care are urgently needed to address these deficiencies and improve outcomes.

## 1. Introduction

Thyroid cancer is recognized as one of the most common endocrine malignancies, currently ranking as the tenth most frequent cancer worldwide [1]. In Saudi Arabia, the incidence of thyroid cancer has significantly increased over the past decades, making it the third most prevalent malignancy in the country, with an overall incidence rate of 10.1%. Although thyroid cancer generally has a favorable prognosis, it still contributes to cancer-related mortality, accounting for a measurable portion of cancer deaths in the population [2]. Notably, there is a pronounced gender disparity, consistent with global trends, with incidence rates significantly higher among females (14.1%) compared to males (6%) in Saudi Arabia [2,3]. Globally, thyroid cancer is approximately three times more common in women than men, a pattern observed across diverse populations and regions [4].

Thyroid cancer includes several histological subtypes, such as papillary thyroid carcinoma, follicular thyroid carcinoma, Hürthle cell carcinoma, medullary thyroid carcinoma, and anaplastic thyroid carcinoma [5]. Among these, papillary thyroid carcinoma dominates, accounting for approximately 80–85% of all thyroid malignancies [1,5]. The etiology of thyroid cancer is multifactorial, involving both modifiable and non-modifiable risk factors. Modifiable risk factors encompass exposure to ionizing radiation, smoking, pre-existing benign thyroid disorders, iodine deficiency or excess, obesity, alcohol consumption, and chronic stress [6]. Non-modifiable factors mainly involve family history and genetic predisposition [5,7]. Environmental exposures, such as radiation during childhood, have been particularly linked to the development of papillary thyroid carcinoma, with risk increasing with higher radiation doses and younger age at exposure [5].

Management of thyroid cancer is largely determined by the histological subtype and disease stage. For differentiated thyroid carcinoma, which includes papillary and follicular types, surgical intervention remains the cornerstone of treatment. Surgical options range from lobectomy or partial thyroidectomy for tumors smaller than 4 cm to near-total or total thyroidectomy, with or without lymph node dissection, for more advanced disease [1]. Additional therapeutic modalities may include radioactive iodine ablation, thyroid hormone suppression therapy, targeted molecular therapies, and, in selected cases, external beam [8]. The prognosis for most thyroid cancers, particularly DTC, is favorable with appropriate management, though aggressive subtypes such as ATC and advanced MTC are associated with poorer outcomes [9].

Understanding the epidemiological trends and risk factor profiles of thyroid cancer in specific populations is essential for guiding public health strategies and shaping healthcare policy [5]. Cross-sectional studies have revealed significant regional variations in public awareness, risk factor recognition, and knowledge of preventive measures, highlighting the need for region-specific assessments of public knowledge, awareness, and understanding of thyroid cancer risk factors, symptoms, and preventive practices [10]. For example, a recent survey by Qusty et al. demonstrated that higher awareness scores were associated with the female gender; however, substantial knowledge gaps persisted among Saudi adults regarding thyroid cancer [11]. Similarly, another study in a different region of Saudi Arabia reported that only 7.7% of female participants exhibited good knowledge of thyroid cancer [12].

Despite the increasing incidence and significant gender disparity of thyroid cancer in Saudi Arabia, comprehensive data on public awareness and knowledge levels, particularly in the Northern region, remain limited. This gap is concerning, as early detection is closely linked to improved outcomes, and public awareness is a key factor in encouraging timely healthcare-seeking behavior [13]. Furthermore, northern Saudi Arabia’s unique cultural, environmental, and demographic characteristics may influence disease patterns and public health knowledge in ways that differ from other regions. This region is characterized by a more dispersed population, with a significant proportion residing in rural or remote areas, which can limit access to specialized healthcare services and preventive programs [14]. Traditional cultural and religious values, including strong community ties and the influence of local leaders, play a significant role in shaping health beliefs and healthcare-seeking behaviors [15]. These factors, combined with variations in climate and lifestyle, contribute to differences in health awareness and access compared to more urbanized regions of the Kingdom [16].

This study aims to assess the knowledge, awareness, and perceived prevalence of thyroid cancer among the general population in the northern region of Saudi Arabia. By identifying specific knowledge gaps and misconceptions, the findings of this study will provide valuable insights to guide the development of targeted educational interventions and screening initiatives tailored to the needs of this population. Specifically, our study population comprises residents of the Northern Border Province of Saudi Arabia, which has a population of approximately 373,577 people distributed across urban centers such as Arar, Rafha, and Turaif, as well as rural communities [16].

## 2. Materials and Methods

### 2.1. Study Design and Setting

This cross-sectional survey was conducted from July to December 2024 in the northern region of Saudi Arabia, encompassing the Arar (the capital of the Northern Border area), Aljouf, Tabuk, and Hail provinces, as described in our previously published work [16]. This study employed a quantitative approach to systematically assess the knowledge and awareness of thyroid cancer among the general population in this region. A quantitative, cross-sectional survey design was chosen because it enables the efficient collection and analysis of data from a large, geographically dispersed population. This approach is particularly well-suited for estimating the prevalence of knowledge gaps and identifying sociodemographic correlates, providing a robust baseline for future targeted interventions and longitudinal studies [17].

### 2.2. Sample Size Calculation and Sampling Technique

The sample size was determined based on the 2022 census population of 1,965,025 for the northern region. Assuming a 50% expected response distribution, a 95% confidence level, and a 5% margin of error, the minimum required sample size was calculated to be 385 participants using the Raosoft sample size calculator (Raosoft, Inc., Seattle, WA, USA). A larger sample was targeted to increase statistical power and compensate for potential incomplete responses. Participants were recruited using a convenience sampling strategy via online platforms and social media channels, aiming to capture a broad and diverse segment of the population. The convenience sampling approach was employed to facilitate timely and efficient data collection, given the logistical and resource constraints associated with reaching a geographically dispersed population in the northern region. While this method enabled broad participation through community centers and online dissemination, the authors acknowledge that it may introduce selection bias, favoring those with greater internet access/digital literacy and limiting the generalizability of the findings.

### 2.3. Participant Recruitment

Participants were recruited using a convenience sampling strategy via widely used online platforms and social media channels, including WhatsApp, Twitter (X), and Facebook. Study invitations containing a brief description of the research objectives, eligibility criteria, and a secure link to the online questionnaire were disseminated through community groups, university networks, and local organizations in the northern region. Participation was voluntary, and all respondents provided informed consent prior to beginning the survey. Inclusion criteria were age 18 years or older, residency in the northern region of Saudi Arabia, and ability to read and understand Arabic. To maximize reach and diversity, reminders were posted at different times and days over four weeks.

### 2.4. Data Collection Instrument and Validity Assessment

Data were collected using a structured questionnaire adapted from a previously validated instrument with modifications to reflect regional thyroid cancer epidemiology and sociocultural context [12] (Supplementary File S1). Content validity was assessed through a two-stage process. First, an expert panel review comprising three independent experts (an endocrine surgeon, an epidemiologist, and a public health specialist) evaluated the questionnaire using a 4-point Likert scale for relevance, clarity, and comprehensiveness. Items achieving a Content Validity Index (CVI) ≥ 0.78 were retained. Second, a pilot test was conducted by administering the revised questionnaire to 20 participants selected to reflect the diversity of the target population in terms of age, gender, education level, and urban/rural residence. The same inclusion and exclusion criteria as the main study were applied to the pilot group, ensuring representativeness and feasibility. This strategy allowed us to assess the clarity, relevance, and comprehensibility of the instrument across key demographic subgroups and to make any necessary adjustments before full deployment [18]. Internal consistency was assessed using Cronbach’s alpha, yielding values of α = 0.81 for knowledge items and α = 0.76 for perception items, indicating acceptable reliability. Ambiguous items (e.g., “chronic stress” as a risk factor) were rephrased based on pilot feedback.

The questionnaire was developed in Google Forms (Google, Inc., Mountain View, CA, USA) and disseminated through various social media platforms to enhance reach and diversity. Of the 1250 individuals contacted via social media platforms (Twitter, WhatsApp, and regional health forums), 702 completed the survey, yielding a 56.2% response rate. These individuals were approached through a combination of targeted posts in public community groups, university and professional networks, and direct messages sent to members of relevant online forums. Each invitation included a brief description of the study, eligibility criteria, and a secure link to the online questionnaire. Reminders were sent at three-day intervals to maximize response rates.

The questionnaire comprised five sections: (1) sociodemographic information (age, gender, nationality, education, occupation, and residence); (2) prevalence and screening practices (personal/family history, screening frequency, and familiarity with screening methods); (3) general awareness and perceptions (knowledge of symptoms, perceived disease severity, and information sources); (4) risk factor knowledge (understanding of 2.5 radiation exposure, genetic predisposition, family history, and lifestyle factors); and (5) diagnostic and treatment awareness (knowledge of diagnostic modalities and available treatments).

### 2.5. Statistical Analysis

Data were entered into Microsoft Excel (Microsoft Corp., Redmond, WA, USA) and analyzed using IBM SPSS Statistics for Windows, version 27 (IBM SPSS Statistics, Armonk, NY, USA). Descriptive statistics were used to summarize sociodemographic characteristics and responses to knowledge and awareness items. Categorical variables were expressed as frequencies and percentages. The chi-square test was employed to assess associations between categorical variables, with statistical significance set at *p* ≤ 0.05. Multivariable logistic regression identified predictors of “good knowledge” (dependent variable), adjusting for age, gender, education, and occupation. Knowledge scores were computed and normalized using Bloom’s cut-off points (poor: ≤60% correct answers, moderate: 61–80%, and good: ≥81% [3].

### 2.6. Ethical Considerations

The study protocol was approved by the local bioethics committee of the Northern Border University (approval number 72/24/H) (5 June 2024). All participants received detailed information about the study objectives, procedures, potential risks, and benefits on the first page of the online questionnaire. Informed consent was obtained electronically: participants were required to read the consent statement and indicate their agreement by selecting a mandatory checkbox before they could proceed to the survey questions. Only those who provided electronic consent were able to access and complete the questionnaire. Participation was voluntary, with the option to withdraw at any time without penalty.

### 2.7. Data Storage and Security

All survey responses were stored securely on encrypted Google Drive cloud storage, accessible only to the principal investigators through password-protected accounts. Data transmission and storage complied with institutional data protection guidelines, and no personal identifiers were collected. Regular backups were performed, and all data were retained solely for research purposes.

## 3. Results

### 3.1. Sociodemographic Characteristics

A total of 702 participants who fully completed the survey were included in the analysis (Table 1). Partial or incomplete responses were excluded from the final dataset. The participants were predominantly young, with over half (50.4%) aged 20–30 years and a further 34.6% aged 31–40 years. Female participants constituted 62% of the group. The vast majority were Saudi nationals (97.3%), and nearly half resided in the Northern Border Province (48.6%), with the remainder distributed across Aljouf (20.5%), Tabuk (16.1%), and Hail (14.8%). Educational attainment was high, with 74.6% holding a bachelor’s degree and 7.5% possessing postgraduate qualifications. Notably, 83.6% reported having family members or friends in the medical field. Regarding healthcare utilization, 40.9% visited a health center more than twice per year. In this study, a “healthcare center” refers primarily to the Ministry of Health primary healthcare centers, which are community-based facilities providing general medical care, preventive services, and referrals to specialist care as needed [19].

### 3.2. General Awareness and Perceptions of Thyroid Cancer

About 45% of participants believed that the disease was curable, while nearly half (49%) were unsure. A majority correctly identified that thyroid cancer is not contagious (72.9%) and is more common in females (51.4%), though substantial uncertainty remained regarding disease curability and prevalence (Table 2). Awareness of the benefits of early detection was relatively high, as 75.6% agreed that early detection enables appropriate treatment, while only 2.7% disagreed and 21.7% were uncertain. Despite the current findings, engagement with thyroid cancer awareness campaigns was low, with only 18.4% having attended or watched such events, underscoring the need for enhanced public health outreach (Table 2).

### 3.3. Screening Practices and Risk Factor Knowledge

A very small minority (1.3%) reported a personal history of thyroid cancer (Table 3). Regarding proactive health management, among all participants, 45.4% had undergone thyroid hormone analysis, while only 9.0% had received thyroid imaging (ultrasound or CT scan). The majority (54.6%) had never had a thyroid hormone test, indicating a gap in routine screening practices. It should be noted that thyroid hormone testing and imaging may be conducted for a variety of reasons, such as evaluation of general symptoms, routine health checks, or as part of other medical investigations, not solely for diagnosed thyroid cancer.

Knowledge of symptoms was moderate, with 64.1% recognizing neck lumps and 56.7% identifying dysphagia as potential indicators (Supplementary Table S1).

### 3.4. Sociodemographic Correlates

Significant associations were observed between thyroid hormone analysis and age, marital status, region, education, and medical field exposure (all *p* < 0.05, Table 4).

Regarding the belief in thyroid cancer prevention, it showed significant associations with nationality (*p* = 0.021) and having family or friends in the medical field (*p* = 0.016) (Table 5). However, as the vast majority of participants were Saudi nationals (97.3%), the association with nationality should be interpreted with caution due to the very small number of non-Saudi respondents.

## 4. Discussion

This cross-sectional study provides the first comprehensive assessment of thyroid cancer awareness, risk factor knowledge, and preventive practices among residents of northern Saudi Arabia. The results reveal substantial gaps in awareness and engagement in preventive behaviors despite a generally high educational level and strong ties to the medical field among participants. Most notably, nearly half of the participants had never undergone thyroid hormone analysis, and over 90% had not received thyroid imaging, indicating limited engagement in routine screening. It should be noted that thyroid hormone analysis is not universally recommended as a routine screening test for all individuals. In the Saudi context, however, targeted screening is advised for individuals with symptoms or risk factors for thyroid dysfunction, as per national and regional guidelines [20]. Therefore, the low rates of thyroid hormone testing and imaging observed in our sample may reflect limited awareness or engagement in preventive health practices among at-risk individuals rather than a deviation from universal screening guidelines. This interpretation is specific to the Saudi healthcare context and may not be generalizable to countries where routine thyroid testing is not part of standard preventive care. Misconceptions about the curability and genetic basis of thyroid cancer were widespread, with only 27.8% recognizing its genetic component and almost half uncertain about prognosis. While a majority believed that thyroid cancer could be prevented, only a minority (18.4%) had participated in awareness campaigns, highlighting a disconnect between knowledge and preventive action.

These findings are consistent with previous studies from other regions of Saudi Arabia, which have also reported low screening rates and limited awareness of thyroid cancer risk factors and symptoms. For example, Qusty et al. [11] and Alyahya et al. [21] both documented substantial knowledge gaps and misconceptions regarding thyroid cancer among Saudi adults. Similarly, studies in Riyadh and the Eastern Province found that awareness of genetic risk and the importance of early detection was limited, and participation in awareness campaigns was low [21,22,23]. However, our study adds to the literature by highlighting the unique context of the northern region, where a high proportion of participants had a university education and connections to the medical field, yet preventive practices and campaign engagement remained suboptimal. This suggests that educational attainment and medical field exposure alone may not be sufficient to drive preventive behaviors or participation in health promotion activities [24].

Importantly, beyond its geographic location, the northern region of Saudi Arabia is demographically and culturally unique. Our sample revealed a notably high proportion of university-educated participants (74.6%), and a striking 83.6% reported having family or friends in the medical field, a distinctive characteristic compared to other regions. Despite this, participation in thyroid cancer awareness campaigns was low (18.4%), indicating a disconnect between educational attainment, informal access to medical knowledge, and engagement in formal health promotion activities. This trend may reflect the region’s scattered population, differences in healthcare infrastructure, and the influence of local cultural norms. The relatively high digital literacy and use of online platforms in this area, as evidenced by our data collection method, also highlight opportunities for digital health interventions.

The observed associations between knowledge or screening practices and factors such as age, education, nationality, and having family or friends in the medical field are in line with previous research [20,25]. Notably, the strong association with medical field connections suggests a potential opportunity for peer-led or community-based interventions leveraging these networks. However, the association with nationality should be interpreted with caution due to the very small number of non-Saudi participants in our sample, as discussed in Section 5.

Given the unique regional characteristics, several practical and context-specific strategies are warranted. (1) Implementing health education programs in schools and universities could be particularly impactful due to the young age and high educational attainment of the population. (2) Integrating thyroid screening, such as hormone analysis or ultrasound, into routine primary healthcare visits is feasible, as 40.9% of participants reported frequent visits to health centers. (3) Leveraging social media and digital platforms for awareness campaigns would likely be effective, considering the high digital engagement in this region. (4) Engaging community and religious leaders in awareness efforts, especially in rural and hail-affected areas, may enhance outreach and credibility. (5) Mobilizing participants with connections to the medical field as peer educators or community ambassadors could further amplify the reach of educational initiatives.

The high digital literacy and use of online platforms observed in our sample indicate that digital and social media campaigns could be particularly effective in reaching this population. Additionally, the low rates of screening and awareness campaign participation suggest that efforts should focus not only on increasing knowledge but also on motivating and enabling preventive behaviors.

To ensure sustainable improvement, future research should include longitudinal and interventional studies to evaluate the effectiveness of these targeted strategies over time. Such studies would build on our cross-sectional findings and provide evidence for scalable, regionally tailored public health policies.

In summary, our study addresses a major gap in the literature by providing the first comprehensive assessment of thyroid cancer awareness in northern Saudi Arabia and by highlighting practical, context-specific recommendations for improving public health outcomes in this region.

## 5. Study Limitations

This study has several limitations that should be considered when interpreting the findings. First, our reliance on self-reported data introduces potential recall bias, reporting bias, and social desirability bias, particularly concerning screening behaviors such as thyroid hormone analysis or imaging. Participants may have over-reported healthy behaviors or under-reported knowledge gaps due to perceived social expectations. Second, the absence of objective verification (e.g., medical records or physician confirmation) limits the accuracy of reported prevalence and preventive practices, potentially leading to underestimation or overestimation of true awareness and engagement, especially among subgroups with lower health literacy or limited healthcare access. Third, using convenience sampling via social media may have introduced selection bias, skewing the sample toward younger, more educated, or health-conscious individuals and limiting the generalizability of our findings to the broader population of northern Saudi Arabia. Fourth, the demographic distribution was not proportionally stratified, which may affect the representation of certain subpopulations. In particular, the number of non-Saudi participants was very small (2.7%), which limits the interpretability and generalizability of any observed associations with nationality. Findings related to nationality should, therefore, be interpreted with caution, and future studies should aim for greater diversity in the sample. Fifth, while our adapted questionnaire was validated, it may not have fully captured culturally specific dimensions of thyroid cancer knowledge relevant to this region. Finally, the lack of qualitative data limited our ability to explore underlying reasons for knowledge gaps and barriers to preventive care.

Future studies should consider integrating mixed-methods approaches, such as using validated clinical records and qualitative interviews, to triangulate findings and reduce exclusive reliance on self-reports. Additionally, incorporating standardized knowledge assessment tools with clearly defined scoring systems may enhance the reliability and comparability of future research.

## 6. Conclusions and Future Perspectives

In summary, this study identifies substantial gaps in thyroid cancer awareness, including limited knowledge of risk factors, insufficient understanding of recommended screening practices, and misconceptions about disease prognosis among residents of northern Saudi Arabia. While the vast majority of participants (98.7%) reported no personal history of thyroid cancer, nearly half had never undergone thyroid hormone analysis, and over 90% had not received thyroid imaging. Misconceptions about prognosis were common, with almost half uncertain about the disease’s curability. Despite a generally high educational level, only a minority (18.4%) had participated in awareness campaigns, highlighting a disconnect between knowledge and preventive action.

These findings underscore the urgent need for comprehensive, culturally tailored educational initiatives and community-based interventions. Given the high digital literacy and active use of online platforms among our participants, as reflected in our recruitment strategy and response rates, integrating thyroid health education into primary care and leveraging digital platforms could be particularly effective in this region. Additionally, engaging local leaders may help to reach underserved or rural populations, as traditional campaign participation was low despite strong ties to the medical field. Future research should focus on evaluating the effectiveness of targeted interventions, using mixed-methods designs to address the low participation in awareness campaigns observed in this study while also exploring the influence of cultural and socioeconomic factors that may underpin the knowledge–behavior gap identified in our findings. Addressing these knowledge gaps, specifically the limited awareness of thyroid cancer risk factors, insufficient understanding of appropriate screening practices, and misconceptions about disease prognosis through strategic public health initiatives, can foster improved awareness, encourage timely healthcare-seeking, and ultimately contribute to better thyroid cancer outcomes in this region.

## Figures and Tables

**Table 1:** Sociodemographic characteristics of participants (*n* = 702).

Parameter		No.	Percent (%)
Age	20–30	354	50.4
	31–40	243	34.6
	41–50	80	11.4
	51–60	16	2.3
	Above 60	9	1.3
Sex	Female	435	62.0
	Male	267	38.0
Nationality	Saudi	683	97.3
	Non-Saudi	19	2.7
Marital status	Single	342	48.7
	Married	342	48.7
	Other	18	2.6
Which region do you currently live in?	Northern border	341	48.6
	Aljouf	144	20.5
	Tabuk	113	16.1
	Hail	104	14.8
Do you have any family members or friends who work in the medical field?	No	115	16.4
	Yes	587	83.6
Education level	High school or below	125	17.8
	Bachelor	524	74.6
	Postgrad education	53	7.5
How often do you visit a health center per year?	None	129	18.4
	Once	138	19.7
	Twice	148	21.1
	More than twice	287	40.9

Data are represented as frequencies (No.: numbers) and percentages (%).

**Table 2:** Participants’ general perception and awareness of thyroid cancer (*n* = 702).

Parameter		No.	Percent (%)
Is thyroid cancer incurable?	Yes	46	6.6
	No	315	44.9
	I do not know	341	48.6
Is thyroid cancer contagious?	Yes	11	1.6
	No	512	72.9
	I do not know	179	25.5
Can thyroid cancer be prevented?	Yes	436	62.1
	No	29	4.1
	I do not know	237	33.8
Is thyroid cancer uncommon in Saudi Arabia?	Yes	94	13.4
	No	232	33.0
	I do not know	376	53.6
Is thyroid cancer more common in males than females?	Males	39	5.6
	Females	361	51.4
	I do not know	302	43.0
Thyroid cancer is more common in those who are older than 40 years	Yes	280	39.9
	No	78	11.1
	I do not know	344	49.0
When thyroid cancer is detected early, it can be treated appropriately and adequately	Yes	531	75.6
	No	19	2.7
	I do not know	152	21.7
Have you ever attended or watched the effectiveness of a particular awareness campaign for thyroid cancer?	Yes	129	18.4
	No	470	67.0
	I do not know	103	14.7

**Table 3:** Parameters related to the prevalence and practices for detecting thyroid cancer according to the participants (*n* = 702).

Parameter		No.	Percent (%)
Have you ever had a history of thyroid cancer?	No	693	98.7
	Yes	9	1.3
Have you ever done a thyroid hormone analysis?	No	383	54.6
	Yes	319	45.4
Have you ever undergone an ultrasound or CT scan of the thyroid gland?	No	639	91.0
	Yes	63	9.0

Data are represented as frequencies (No.: numbers) and percentages (%).

**Table 4:** The relationship between undergoing a thyroid hormone analysis and sociodemographic characteristics.

Parameters		Have You Ever Done a Thyroid Hormone Analysis?		Total (*n* = 702)	*p*-Value
		No	Yes		
Sex	Female	226	209	435	0.077
		59.0%	65.5%	62.0%	
	Male	157	110	267	
		41.0%	34.5%	38.0%	
Age	20–30	243	111	354	**0.0001**
		63.4%	34.8%	50.4%	
	31–40	100	143	243	
		26.1%	44.8%	34.6%	
	41–50	30	50	80	
		7.8%	15.7%	11.4%	
	51–60	6	10	16	
		1.6%	3.1%	2.3%	
	Above 60	4	5	9	
		1.0%	1.6%	1.3%	
Nationality	Saudi	368	315	683	**0.030**
		96.1%	98.7%	97.3%	
	Non-Saudi	15	4	19	
		3.9%	1.3%	2.7%	
Marital status	Single	234	108	342	**0.0001**
		61.1%	33.9%	48.7%	
	Married	134	208	342	
		35.0%	65.2%	48.7%	
	Other	15	3	18	
		3.9%	0.9%	2.6%	
Residential region	Northern borders	169	172	341	**0.001**
		44.1%	53.9%	48.6%	
	Aljouf	91	53	144	
		23.8%	16.6%	20.5%	
	Tabuk	74	39	113	
		19.3%	12.2%	16.1%	
	Hail	49	55	104	
		12.8%	17.2%	14.8%	
Education level	High school or below	86	39	125	**0.002**
		22.5%	12.2%	17.8%	
	Bachelor	268	256	524	
		70.0%	80.3%	74.6%	
	Postgrad education	29	24	53	
		7.6%	7.5%	7.5%	
Do you have any family members or friends who work in the medical field?	No	74	41	115	**0.021**
		19.3%	12.9%	16.4%	
	Yes	309	278	587	
		80.7%	87.1%	83.6%	
How often do you visit a health center per year?	None	85	44	129	**0.014**
		22.2%	13.8%	18.4%	
	Once	77	61	138	
		20.1%	19.1%	19.7%	
	Twice	81	67	148	
		21.1%	21.0%	21.1%	
	More than twice	140	147	287	
		36.6%	46.1%	40.9%	

Data are represented as frequencies (No.: numbers) and percentages (%). The chi-square test or Fisher’s exact test was applied where appropriate. Bold values indicate significance at *p*-values ≤ 0.05.

**Table 5:** Participants’ responses regarding “If thyroid cancer can be prevented” and sociodemographic characteristics.

Parameters		Can Thyroid Cancer Be Prevented?		Total (*n* = 702)	*p*-Value
		No, or I Do Not Know	Yes		
Sex	Female	157	278	435	0.210
		59.0%	63.8%	62.0%	
	Male	109	158	267	
		41.0%	36.2%	38.0%	
Age	20–30	130	224	354	0.751
		48.9%	51.4%	50.4%	
	31–40	96	147	243	
		36.1%	33.7%	34.6%	
	41–50	32	48	80	
		12.0%	11.0%	11.4%	
	51–60	4	12	16	
		1.5%	2.8%	2.3%	
	Above 60	4	5	9	
		1.5%	1.1%	1.3%	
Nationality	Saudi	254	429	683	**0.021**
		95.5%	98.4%	97.3%	
	Non-Saudi	12	7	19	
		4.5%	1.6%	2.7%	
Marital status	Single	126	216	342	0.520
		47.4%	49.5%	48.7%	
	Married	135	207	342	
		50.8%	47.5%	48.7%	
	Other	5	13	18	
		1.9%	3.0%	2.6%	
Residential region	Northern border	128	213	341	0.560
		48.1%	48.9%	48.6%	
	Aljouf	61	83	144	
		22.9%	19.0%	20.5%	
	Tabuk	42	71	113	
		15.8%	16.3%	16.1%	
	Hail	35	69	104	
		13.2%	15.8%	14.8%	
Education level	High school or below	56	69	125	0.117
		21.1%	15.8%	17.8%	
	Bachelor	187	337	524	
		70.3%	77.3%	74.6%	
	Postgrad education	23	30	53	
		8.6%	6.9%	7.5%	
Do you have any family members or friends who work in the medical field?	No	55	60	115	**0.016**
		20.7%	13.8%	16.4%	
	Yes	211	376	587	
		79.3%	86.2%	83.6%	
How often do you visit a health center per year?	None	58	71	129	0.145
		21.8%	16.3%	18.4%	
	Once	55	83	138	
		20.7%	19.0%	19.7%	
	Twice	57	91	148	
		21.4%	20.9%	21.1%	
	More than twice	96	191	287	
		36.1%	43.8%	40.9%	

Data are represented as frequencies (No.: numbers) and percentages (%). The chi-square test or Fisher’s exact test was applied where appropriate. Bold values indicate significance at *p*-values ≤ 0.05.

## Data Availability

The original contributions presented in this study are included in the article/Supplementary Materials. Further inquiries can be directed to the corresponding author.

## Supplementary Materials

The following supporting information can be downloaded at: https://www.mdpi.com/article/10.3390/healthcare13111289/s1, Table S1: Participants’ awareness of the risk factors, diagnosis, and treatment of thyroid cancer (*n* = 702); File S1: The study questionnaire.
