# Supplementary material for: Exploring the Public Awareness of Thyroid Cancer in Northern Saudi Arabia: A Preliminary Stage for Health Promotion
Source: Healthcare (Basel). 2025 May 29;13(11):1289. doi: 10.3390/healthcare13111289 (PMC12154295; doi:10.3390/healthcare13111289)
Supplement: Supplementary file 1 [file healthcare-13-01289-s001.zip › A1_Questionnaire_Healthcare.pdf]

QUESTIONNAIRE

Public awareness of thyroid cancer in Northern Saudi Arabia

You are invited to participate in this study to determine the public awareness of thyroid cancer in Northern Saudi Arabia

- **Your participation** will include completing a brief questionnaire (about 5-7 minutes).

- **Participation is voluntary**, and you can withdraw from this study anytime.

- **Confidentiality:** All information collected during the study will be kept strictly confidential. Your name and identifying information will not be associated with your responses.

- **The data** will be used for research purposes only and will not be shared with anyone outside the research team.

- **Risks and Benefits:** There are no known risks associated with participating in this study. You may benefit from increased the public awareness of thyroid cancer in Northern Saudi Arabia which may help in doing future initiatives related to this issue.

You are agreeing to participate in this study **Yes**

No

❖ Demographic data

1- Age (years)

2- Nationality
- Saudi
- Non-Saudi

3- Marital status
- Single
- Married
- Divorced
- Widow

4- Do you currently live in the Northern Border region?
- Yes
- No

5- Do you have any family members or friends that work in the medical field?
- Yes
- No

6- Education level
- High school or below
- Bachelor
- Post-grad education

7- How often do you visit a health center per year?
- None
- Once
- Twice
- More than twice

❖ Prevalence and Practices for detecting thyroid cancer according to the participants

- Have you ever had a history of thyroid cancer?
  - Yes
  - No

- Have you ever done a thyroid hormone analysis?
  - Yes
  - No

- Have you ever undergone an ultrasound or CT scan of the thyroid gland?
  - Yes
  - No

❖ General perception and awareness of TC

1- Is thyroid cancer incurable?
- Yes
- No
- I do not know

2- Is thyroid cancer contagious?
- Yes
- No
- I do not know

3- Can thyroid cancer be prevented?
- Yes
- No
- I do not know

4- Thyroid cancer is uncommon in Saudi Arabia?
- Yes
- No
- I do not know

5- Thyroid cancer is more common in (males/females)
- Male
- Female
- I do not know

6- Thyroid cancer is more common in those who are older than 40 years
- Yes
- No
- I do not know

7- When thyroid cancer is detected early, it can be treated appropriately and adequately
- Yes
- No
- I do not know

8- Have you ever attended or watched the effectiveness or special awareness campaign for thyroid cancer?

- Yes
- No

❖ Awareness of the risk factors of TC

9- Is thyroid cancer often genetic?

- Yes
- No
- I do not know

10- Lifestyle is associated with an increased risk of thyroid cancer; for example, stability or diet

- Yes
- No
- I do not know

11- The presence of a risk factor for thyroid cancer means that I am at increased risk of developing the disease

- Yes
- No
- I do not know

12- Does physical activity reduce the risk of thyroid cancer?

- Yes
- No
- I do not know

13- Does obesity increase the risk of thyroid cancer?

- Yes
- No
- I do not know

14- Does radiation exposure increase the risk of thyroid cancer?

- Yes
- No
- I do not know

❖ Awareness of the diagnosis and treatment of TC

15- Thyroid cancer symptoms include a change of voice

- Yes
- No
- I do not know

16- Thyroid cancer symptoms include a dysphasia

- Yes
- No
- I do not know

17- Thyroid cancer symptoms include a dyspnea

- Yes
- No
- I do not know

18- Thyroid cancer appears in the form of a lump or knot in the neck

- Yes
- No
- I do not know

19- Monitoring the presence of swelling in the neck is useful for the early detection of thyroid cancer

- Yes
- No
- I do not know

20- If you find a lump or knot in the thyroid area, you will visit the doctor for a consultation.

- Yes
- No
- I do not know

Thank you
