# Supplementary material for: Exploring the Public Awareness of Thyroid Cancer in Northern Saudi Arabia: A Preliminary Stage for Health Promotion
Source: Healthcare (Basel). 2025 May 29;13(11):1289. doi: 10.3390/healthcare13111289 (PMC12154295; doi:10.3390/healthcare13111289)
Supplement: Supplementary file 1 [file healthcare-13-01289-s001.zip › Table S1.pdf]

Exploring the Public Awareness of Thyroid Cancer in Northern Saudi Arabia: A Preliminary Stage for Health Promotion

Table S1. Participants' awareness of the risk factors, diagnosis, and treatment of thyroid cancer (n = 702).

Parameter		No.	Percent (%)
Is thyroid cancer often genetic?	Yes	195	27.8
	No	157	22.4
	I do not know	350	49.9
Lifestyle is associated with an increased risk of thyroid cancer.	Yes	370	52.7
	No	77	11.0
	I do not know	255	36.3
The presence of a risk factor for thyroid cancer means that I am at increased risk of developing the disease	Yes	141	20.1
	No	217	30.9
	I do not know	344	49.0
Does physical activity reduce the risk of thyroid cancer?	Yes	359	51.1
	No	59	8.4
	I do not know	284	40.5
Does obesity increase the risk of thyroid cancer?	Yes	353	50.3
	No	56	8.0
	I do not know	293	41.7
Does radiation exposure increase the risk of thyroid cancer?	Yes	264	37.6
	No	81	11.5
	I do not know	357	50.9
Thyroid cancer symptoms include a change in voice	Yes	360	51.3
	No	45	6.4
	I do not know	297	42.3
Thyroid cancer symptoms include dysphasia	Yes	398	56.7
	No	36	5.1
	I do not know	268	38.2
Thyroid cancer symptoms include dyspnea	Yes	336	47.9
	No	49	7.0
	I do not know	317	45.2
Thyroid cancer appears in the form of a lump or knot in the neck	Yes	450	64.1
	No	27	3.8
	I do not know	225	32.1
Monitoring the presence of swelling in the neck is helpful for the early detection of thyroid cancer	Yes	485	69.1
	No	29	4.1
	I do not know	188	26.8
If you find a lump or knot in the thyroid area, you should consult a doctor.	Yes	560	79.8
	No	20	2.8
	I do not know	122	17.4

Data are represented as frequencies (No.: numbers) and percentages (%).
